# Risk factors for agitation in critically ill patients

**DOI:** 10.5935/0103-507X.20160074

**Published:** 2016

**Authors:** Thiago Miranda Lopes de Almeida, Luciano Cesar Pontes de Azevedo, Paulo Maurício Garcia Nosé, Flavio Geraldo Resende de Freitas, Flávia Ribeiro Machado

**Affiliations:** 1 Anesthesiology, Pain and Intensive Care Department, Escola Paulista de Medicina, Universidade Federal de São Paulo - Sao Paulo (SP), Brazil.

**Keywords:** Psychomotor agitation, Risk factors, Delirium, Pain, Respiration, artificial, Intensive care, Agitação psicomotora, Fatores de risco, *Delirium*, Dor, Respiração artificial, Cuidados intensivos

## Abstract

**Objective:**

To evaluate the incidence of agitation in the first 7 days after intensive care unit admission, its risk factors and its associations with clinical outcomes.

**Methods:**

This single-center prospective cohort study included all patients older than 18 years with a predicted stay > 48 hours within the first 24 hours of intensive care unit admission. Agitation was defined as a Richmond Agitation Sedation Scale score ≥ +2, an episode of agitation or the use of a specific medication recorded in patient charts.

**Results:**

Agitation occurred in 31.8% of the 113 patients. Multivariate analysis showed that delirium [OR = 24.14; CI95% 5.15 - 113.14; p < 0.001], moderate or severe pain [OR = 5.74; CI95% 1.73 - 19.10; p = 0.004], mechanical ventilation [OR = 10.14; CI95% 2.93 - 35.10; p < 0.001], and smoking habits [OR = 4.49; CI95% 1.33 - 15.17; p = 0.015] were independent factors for agitation, while hyperlactatemia was associated with a lower risk [OR = 0.169; CI95% 0.04 - 0.77; p = 0.021]. Agitated patients had fewer mechanical ventilation-free days at day 7 (p = 0.003).

**Conclusion:**

The incidence of agitation in the first 7 days after admission to the intensive care unit was high. Delirium, moderate/severe pain, mechanical ventilation, and smoking habits were independent risk factors. Agitated patients had fewer ventilator-free days in the first 7 days.

## Abstract (pt)

**Objetivo:**

Avaliar a incidência de agitação nos primeiros 7 dias após admissão à unidade de terapia intensiva, seus fatores de risco e associação com desfechos clínicos.

**Métodos:**

Estudo de coorte unicêntrico prospectivo que incluiu maiores 18 anos, admitidos à unidade de terapia intensiva há menos de 24 horas e com previsão de permanência superior a 48 horas. Agitação psicomotora foi definida como pontuação igual ou superior a +2 na Escala de Agitação e Sedação de Richmond ou episódio de agitação, ou registro de uso de medicação específica na ficha clínica.

**Resultados:**

Ocorreu agitação em 31,8% dos 113 pacientes incluídos. Na análise multivariada, *delirium* (OR = 24,14; IC95% 5,15 - 113,14; p < 0,001), dor moderada ou intensa (OR = 5,74; IC95% 1,73 - 19,10; p = 0,004), ventilação mecânica (OR = 10,14; IC95% 2,93 - 35,10; p < 0,001) e tabagismo (OR = 4,49; IC95% 1,33 - 15,17; p = 0,015) foram independentemente associados a maior risco de desenvolver de agitação. Por outro lado, hiperlactatemia associou-se a um menor risco de ocorrência de agitação (OR = 0,169; IC95% 0,04 - 0,77; p = 0,021). Pacientes agitados tiveram menor tempo livre de ventilação mecânica em 7 dias (p = 0,003).

**Conclusão:**

A incidência de agitação nos 7 primeiros dias de internação em unidade de terapia intensiva foi elevada. *Delirium*, dor moderada ou intensa, ventilação mecânica e tabagismo foram fatores de risco independentes para o desenvolvimento de agitação. Pacientes agitados tiveram menor tempo livre de ventilação mecânica nos 7 primeiros dias.

## INTRODUCTION

Agitation in critically ill patients is a phenomenon that can compromise patient safety and assistance during intensive care unit (ICU) hospitalizations. It is characterized by increased motor and mental activity that manifests as inappropriate behavior, disorganized thoughts and a loss of self-control over actions. Agitation often masks diagnostics, delays treatment onset, which may have an impact on the morbidity and mortality of this population.^(1-3)^

The genesis of agitation is multifactorial.^(4-6)^ Some medical conditions can coexist with or precede agitation episodes. These factors interact with the underlying disease and may increase the occurrence of hyperactivity episodes in the population.^(1,3,7)^ Metabolic demand is increased during periods of agitation, which could compromise energy balance and precipitate organ dysfunction that, in turn, contributes to the loss of homeostasis among critically ill patients.^(1)^ There is also an increased chance of self-extubation, removal of devices, falls and injuries in the presence of agitation.^(8-11)^ Agitation is associated with a longer duration of mechanical ventilation (MV), an increased length of hospital and ICU stay, higher mortality rates and higher costs.^(3,5,8,12-16)^

The assessment of risk factors for agitation among critically ill patients may help understand its genesis and clinical context. This knowledge can provide a foundation for further clinical studies to test therapeutic and preventive strategies for agitation in the context of intensive care. Thus, the objectives of this study were to evaluate the incidence of agitation in the first seven days of intensive care unit admission, to identify the risk factors for its development and to assess its associations with poor clinical evolution.

## METHODS

This was a single-center, prospective cohort study conducted among patients admitted to a 17-bed general ICU at a university hospital. We included patients who were at least 18 years old within the first 24 hours of ICU admission and who had a predicted stay of more than 48 hours. Pregnant women, patients with previous psychiatric conditions, patients transferred from another ICU and those who had used haloperidol, dexmedetomidine, risperidone, or quetiapine prior to the study were excluded. The study was approved by the institution's Research and Ethics Committee (655.838) without the need for informed consent due to its observational nature.

All patients were visited twice daily in the first 7 days of admission. In this prospective assessment, we considered agitated patients to be those with a Richmond Agitation Sedation Scale (RASS) score equal to or greater than +2.^(17)^ We also retrospectively included those who had an episode of agitation recorded in their charts at any time during the day and those who received specific medications for agitation, such as quetiapine, risperidone, haloperidol or dexmedetomidine, which were exclusively used for agitation according to unit standards. All remaining subjects were considered non-agitated.

During the initial visit, we obtained baseline and demographic data as well as information on previous hospital stays, the type and reason for admission, patient origin, Charlson comorbidity index, presence of other comorbidities, Simplified Acute Physiology Score 3 (SAPS 3), and the Sequential Organ Failure Assessment (SOFA).^(18)^ We also recorded the presence of multiple trauma, defined as trauma in more than two organs or systems, and severe brain injury, defined by a Glasgow Coma Score < 8 on arrival at the hospital.

During subsequent visits in the first 7 days, data were recorded on the clinical outcomes and potential risk factors for agitation. We administered the Confusion Assessment Method for Intensive Care Units (CAM-ICU)^(19)^ and an analog pain scale twice a day. Patients with a RASS > 1 and a positive CAM-ICU were considered to have agitation secondary to hyperactive delirium. We considered pain to be moderate/severe when the score was greater than or equal to 3, within a range from 0 to 10. We documented all times when the scales could not be applied because of unresponsiveness. We also collected data on the SOFA score, use of anticholinergic medications,^(20)^ sedatives, opioids or vasopressors, presence of pressure ulcers, sepsis,^(21)^ acute respiratory distress syndrome,^(22)^ hyperlactatemia (lactate > 14mg/dL), fever (axillary temperature > 37.8 °C) and the use of invasive devices. The need for MV and renal replacement therapy were also collected. We also recorded information about the presence of a clock in the room and the frequency of family visits.

Among patients without agitation, the total observation period was 7 days. Among agitated patients, only the variables present before the first episode of agitation were computed. We followed all patients until hospital discharge to assess the pre-defined outcomes. We analyzed ICU-free days and hospital-free days in 28 days, MV-free days and vasopressor-free days in the first 7 days, and ICU and hospital mortality.

### Statistics

The sample size was estimated considering a frequency of agitation of 25% among those exposed to risk factors and 10% among those not exposed. The required size was estimated to be 99 individuals based on a power of 80% and a 5% significance level in a 2-sided hypothesis test.^(23)^ Comparisons of categorical variables were made using chi-square tests. Continuous variables were presented as the mean ± standard deviation or the median (interquartile range) according to the Kolmogorov-Smirnov normality test. We used Student's *t* -test or Mann-Whitney U test to compare the variables between patients with and without agitation as appropriate. We selected variables in the univariate analysis with a p value below 0.05, and those considered clinically relevant were included in the multivariate analysis model using a backward stepwise selection procedure. The results of the multivariate analysis were expressed as odds ratios with 95% confidence intervals. We used Statistical Package for Social Science (SPSS) v. 22.0 for Windows (Chicago, IL, USA) for the statistical analysis. In all analyses, we considered p < 0.05 statistically significant.

## RESULTS

**Figure 1:**
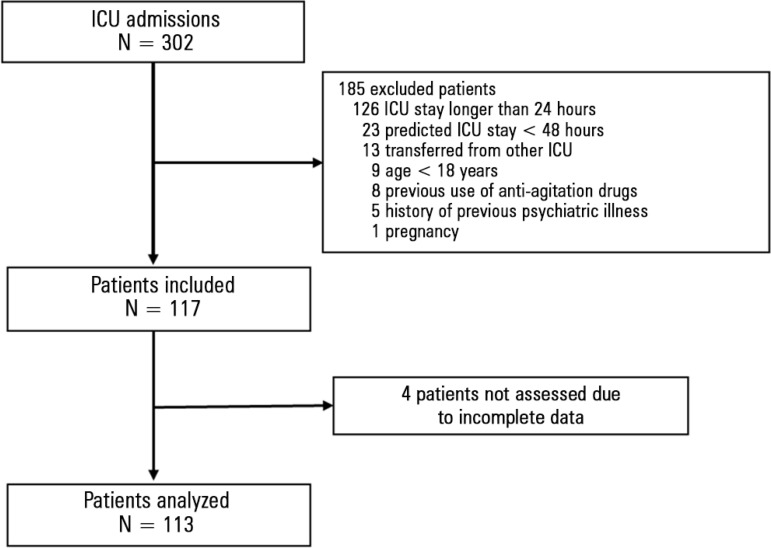
Enrollment flowchart. ICU - intensive care unit.

Between April and August 2014, 302 patients were hospitalized at the ICU. Of these, 185 were excluded; the main reasons for exclusion are depicted in figure 1. We included 117 patients, and 4 were not analyzed due to incomplete data collection. Thus, our sample consisted of 113 patients. Their main baseline characteristics are described in table 1.

**Table 1:** Characteristics of the study population in the entire group according to agitation status

Variables	All patients (N = 113)	Not agitated (N = 77)	Agitated (N = 36)	p value
Age (years)	55.2 ± 18.7	56.3 ± 17.0	52.7 ± 21.8	0.342
Male	63 (55.8)	40 (51.9)	23 (63.9)	0.234
Prior hospital stays (days)	3.0 (2.0 - 10.5)	3.0 (2.0 - 8.0)	2.0 (2.0 - 6.0)	0.777
Type of hospitalization				
Clinic	31 (27.4)	21 (27.3)	10 (27.8)	0.955
Elective surgery	31 (27.4)	22 (28.6)	9 (25.0)	0.691
Urgent surgery	51 (45.1)	34 (44.2)	17 (47.2)	0.760
Location				
Operating room	77 (68.1)	51 (66.2)	26 (72.2)	0.524
Emergency room	19 (16.8)	13 (16.9)	6 (16.7)	0.977
Ward	16 (14.2)	13 (16.9)	3 (8.3)	0.225
Other	1 (0.9)	0	1 (2.8)	0.142
Reason for admission				
Postoperative monitoring	49 (43.4)	36 (46.8)	13 (36.1)	0.288
Sepsis	16 (14.2)	14 (18.2)	2 (5.6)	0.073
Respiratory failure	11 (9.7)	8 (10.4)	3 (8.3)	0.731
Acute neurological disease*	19 (16.8)	7 (9.1)	12 (33.3)	0.001
Multiple trauma	4 (3.5)	4 (5.2)	0	0.164
Other	14 (12.5)	8 (10.4)	6 (16.7)	0.451
SAPS 3 (points)	44.8 ± 15.2	46.2 ± 14.6	41.6 ± 16.3	0.139
SOFA at admission (points)	2.5 (1.0 - 5.2)	4.0 (2.0 - 7.0)	4.0 (2.0 - 7.0)	0.675
Charlson score (points)	4.0 ± 2.9	4.1 ± 2.8	3.9 ± 3.1	0.719
Comorbidities				
Chronic renal failure	14 (12.4)	9 (11.7)	5 (13.9)	0.741
Arterial hypertension	55 (48.7)	36 (46.8)	19 (52.8)	0.551
Hearing impairment	11 (9.7)	5 (6.5)	6 (16.7)	0.089
Visual impairment	41 (36.3)	25 (32.5)	16 (44.4)	0.217
Alcohol abuse	20 (17.7)	10 (13.0)	10 (27.8)	0.055
Tobacco use	23 (20.3)	11 (14.3)	12 (33.3)	0.019
Diabetes mellitus	26 (23.0)	19 (24.7)	7 (19.4)	0.538
COPD	11 (9.7)	7 (9.1)	4 (11.1)	0.736
Severe TBI	11 (9.7)	4 (5.2)	7 (19.4)	0.017
Glasgow at ICU admission	13.5 (10.0 - 14.0)	15.0 (14.0 - 15.0)	13.5 (10.0 - 14.0)	< 0.001
Bed clock absent	83 (73.4)	57 (74)	26 (72.2)	0.840
Delirium	20 (17.7)	4 (5.2)	16 (44.4)	< 0.001
Pain	60 (53.1)	37 (48.1)	23 (63.9)	0.116
Moderate to severe pain	48 (42.5)	27 (35.1)	21 (58.3)	0.020
MV use†	57 (50.4)	30 (39.0)	27 (75.0)	< 0.001
Sepsis	47 (41.6)	35 (45.5)	12 (33.3)	0.223
Vasopressor use†	48 (42.5)	35 (45.5)	13 (36.1)	0.349
Hyperlactatemia	29 (25.7)	24 (31.2)	5 (13.9)	0.050
ARDS	11 (9.7)	10 (13.0)	1 (2.8)	0.088
RRT	11 (9.7)	8 (10.4)	3 (8.3)	0.731
Fever	22 (19.5)	18 (23.4)	4 (11.1)	0.125
Pressure ulcers	5 (4.4)	4 (5.2)	1 (3.2)	0.660
Family absent during visits	35 (31.0)	24 (31.2)	11 (30.6)	0.948
Invasive devices	106 (93.8)	72 (93.5)	34 (94.4)	0.847
Anticholinergic drugs	47 (41.6)	33 (42.9)	14 (38.9)	0.690
Sedatives and opioids	83 (73.4)	57 (76.0)	26 (72.2)	0.668

SAPS - Simplified Acute Physiology Score; SOFA - Sequential Organ Failure Assessment; COPD - chronic obstructive pulmonary disease; TBI - traumatic brain injury; ICU - intensive care unit; MV - mechanical ventilation; ARDS - acute respiratory distress syndrome; RRT - renal replacement therapy.

* Including ischemic or hemorrhagic stroke, subarachnoid hemorrhage, myasthenia and traumatic brain injury;

† considering only patients under mechanical ventilation (N = 57) and vasopressors (N = 48). The results are expressed as the mean ± standard deviation, median (25% - 75%) or number (%). Chi-square or Student’s *t*-tests were used as appropriate.

The incidence of agitation in the first 7 days of ICU hospitalization was 31.8%. The mean time to the onset of agitation was 2.4 ± 1.7 days. Among the agitated patients, the SOFA score on the agitation day was 4.0 (3.0 - 6.0). Pain and delirium could not be assessed in 57.1% and 53% of the attempts because of unresponsiveness. The univariate analysis showed that agitation was more frequent among patients who had a history of smoking, severe head injuries, hospitalization for acute neurological disease, moderate to severe pain, MV, and delirium. On the other hand, agitation was less frequent among patients with hyperlactatemia. There were no associations between the occurrence of agitation and age, severity of disease, SOFA and SAPS 3 scores, or hearing and visual impairment. These data are available in table 1.

The multivariate analysis included variables with a p ≤ 0.05 in the univariate analysis and those that were considered clinically relevant, namely, smoking, alcoholism, delirium, moderate or severe pain, MV and hyperlactatemia. As observed in table 2, the factors independently associated with a higher incidence of agitation were the presence of delirium, moderate or severe pain, MV, and smoking. The presence of hyperlactatemia remained a protective factor for agitation.

**Table 2:** Risk factors for agitation in intensive care unit patients - multivariate analysis

Variable	Odds ratio	95%CI	p value
Smoking habit	4.49	1.33 - 15.17	0.015
Delirium	24.14	5.15 - 113.14	< 0.001
Moderate or severe pain	5.74	1.73 - 19.10	0.004
Mechanical ventilation	10.14	2.93 - 35.10	< 0.001
Hyperlactatemia	0.169	0.04 - 0.77	0.021

95%CI - 95% confidence interval. Backward stepwise selection procedure was used for the logistic regression - likelihood ratio. Hosmer and Lemeshow test: p = 0.102.

Agitated patients had fewer MV free-days and lower hospital mortality than non-agitated patients (Table 3). However, after adjusting for age and SAPS 3 score, MV free-days remained significantly associated with the presence of agitation only, and hospital mortality was no longer significant [odds ratio 3.01; CI95% 0.89 - 10.26; p = 0.770].

**Table 3:** Hospital outcomes according to agitation status

Variables	Not agitated (N = 77)	Agitated (N = 36)	p value
ICU-free days in 28 days	22.0 (11.5 - 24.5)	20.0 (12.0 - 23.0)	0.226
Hospital-free days in 28 days	9.0 (0 - 19.0)	11.0 (0 - 18.7)	0.228
MV-free days in 7 days	7.0 (3.5 - 7.0)	5.0 (1.2 - 6.7)	0.003
Vasopressor-free days in 7 days	7.0 (5.0 - 7.0)	7.0 (5.0 - 7.0)	0.495
ICU mortality	13 (17.1)	3 (8.3)	0.215
Hospital mortality	21 (28.4)	4 (11.1)	0.043

ICU - intensive care unit; MV - mechanical ventilation. Results are expressed as the number (%), mean ± standard deviation or median (25% - 75%). Chi-square or Student’s *t*-tests were used as appropriate.

## DISCUSSION

In this study, we found a high incidence of agitation in the first 7 days of ICU admission. In most cases, patients experienced agitation in the first 3 days after admission, and the factors associated with its occurrence were the presence of delirium, moderate or severe pain, MV, and a smoking habit. Patients with hyperlactatemia had a lower incidence of agitation. Agitated patients had fewer MV free-days.

The incidence of agitation in our study was lower than those previously reported in similar populations. Jaber et al. reported an agitation incidence of 52%,^(5)^ while an even higher incidence (70%) was found by Fraser et al.^(6)^ A higher incidence has also been reported in studies including patients under prolonged MV^(5)^ and in critically ill clinical patients.^(9)^ This variation may be due to differences in the criteria used to define agitation and the use of different diagnostic tools, as well as a longer observation period after ICU admission.

As expected, delirium was an independent risk factor for agitation in the first 7 days of ICU admission. In this time window, delirium occurred in 17.7% of the patients. This incidence was lower than that of other studies in critically ill patients because of our shorter duration of observation. Delirium is a highly prevalent condition in critically ill patients (20 - 80%).^(22-30)^ Peterson et al.^(30)^ reported a 71.5% prevalence of delirium, of which 54.9% were mixed type, showing that patients in ICUs frequently have moments of hyperactivity.^(8,29-31)^

However, we were able to identify other risk factors for agitation that were not related to the presence of delirium. This is a relevant finding, as a misdiagnosis of delirium can lead to inadequate treatment for both the underlying cause and for delirium itself. A previous habit of smoking is recognized as a risk factor for agitation, given the risk of withdrawal syndrome.^(32,33)^ Lucidarme et al.,^(32)^ in a study that included predominantly critically ill medical patients, showed that smokers had a higher incidence of agitation than non-smokers. Moderate or severe pain was more common among agitated patients. The majority of our studied patients were surgical (72.5%), which means that they had high exposures to pain in the first 7 days of observation. Previous studies that showed an association between pain and agitation did not assess whether the patient's pain occurred before agitation.^(13,34-38)^ In our study, we clearly showed that pain is a risk factor of agitation, as only episodes occurring before agitation were considered. MV was also associated with a higher risk of agitation, as previously reported by Woods et al.^(9)^ Potential reasons for this association include the presence of the endotracheal tube, respiratory secretions and asynchrony with the ventilator. Patients under MV might not be able to communicate their needs to the healthcare team. The inability to communicate has previously been described as a risk factor for agitation.^(11)^ In our unit, sedation was maintained as minimal as possible. Our finding suggests that the current no sedation or minimal sedation protocols need to also include a frequent assessment of pain and discomfort among patients using endotracheal tubes and MV.^(39)^

An unexpected finding was the lower incidence of agitation among patients with hyperlactatemia. Although we did not assess the potential mechanisms associated with this relationship, we can hypothesize that patients who develop tissue dysoxia may be more severely ill than those without signs of abnormal cellular metabolism.^(18)^ More severe patients might require continuous long-term sedation, which can contribute to a lower incidence of agitation.^(11)^ Another potential reason is the presence of neurological impairment or renal or hepatic dysfunction that could lead to a reduction in the level of consciousness, limiting the occurrence of agitation. The presence of neuromuscular weakness might also limit the clinical manifestation of agitation.

We were unable to show an association between age and agitation. Although age has been considered a risk factor for agitation, recent prospective studies have shown that age is a protective factor.^(5,6,9)^ As delirium is frequent among agitated patients and among the elderly, it is possible that the prevalence of the hypoactive subtype among patients older than 65 years influences the potential association between age and agitation.^(30)^ We were also not able to show an association between alcohol abuse and agitation. This relationship was expected, as abstinence is a well-known risk factor for agitation. The lack of association might be a consequence of the low prevalence of alcohol abuse among our patients.

Similar to other studies,^(5,9)^ agitated patients had a longer duration of MV in the first 7 days, although no difference was found in hospital mortality.^(5,9)^ We were unable to show an association between agitation and increased use of sedatives or higher severity of illness, which could possibly explain this finding. However, we can hypothesize that being agitated might have precluded attempts to discontinue MV, as suggested by others.^(1)^

This study has some strengths. First, we were able to prospectively determine the moment of agitation, and we were thus able to collect all data regarding risk factors before its occurrence. We consecutively followed all patients admitted to the ICU using a very careful assessment. However, it is worth highlighting some limitations. First, although we included the planned number of patients, studies with small sample sizes are subject to bias. Second, the consecutiveness of the inclusion procedure may have been compromised, as a third of the patients were excluded because they had been admitted for more than 24 hours, mostly on the weekends when the study team was not always available. This also led to a high incidence of missing data among the included patients. Third, the high frequency of MV also compromised the pain and delirium assessments. Fourth, we did not collect data on the presence of agitation during the patients' entire ICU stay, which may have reduced our incidence of agitation. We also prospectively evaluated the presence of agitation only twice per day. The assessment of the entire day was conducted in a retrospective manner, and cases might have been missed. Additionally, we used the administration of antipsychotic drugs to define the presence of agitation. Although the use of these drugs is well controlled in our unit, misuse for other indications might have occurred. Finally, we did not collect data on agitation treatment, which might have influenced the outcome. However, this was not one of our objectives.

The results reinforce the fact that in addition to delirium, there are other independent risk factors for agitation among ICU patients. Good care practices, sedation, analgesia, and management of MV could reduce the incidence of agitation and provide benefits to patients admitted to the ICU.^(11,20,40-45)^

## CONCLUSION

Agitation in the first 7 days of intensive care unit admission was common. The incidence of delirium, moderate or severe pain, mechanical ventilation, and smoking were independent risk factors for the development of agitation. The presence of agitation was associated with fewer mechanical ventilation-free days.
